# Linking gut microbiota, metabolic syndrome and economic status based on a population-level analysis

**DOI:** 10.1186/s40168-018-0557-6

**Published:** 2018-09-24

**Authors:** Yan He, Wei Wu, Shan Wu, Hui-Min Zheng, Pan Li, Hua-Fang Sheng, Mu-Xuan Chen, Zi-Hui Chen, Gui-Yuan Ji, Zhong-Dai-Xi Zheng, Prabhakar Mujagond, Xiao-Jiao Chen, Zu-Hua Rong, Peng Chen, Li-Yi Lyu, Xian Wang, Jia-Bao Xu, Chong-Bin Wu, Nan Yu, Yan-Jun Xu, Jia Yin, Jeroen Raes, Wen-Jun Ma, Hong-Wei Zhou

**Affiliations:** 1 State Key Laboratory of Organ Failure Research, Microbiome Medicine Center, Division of Laboratory Medicine, Zhujiang Hospital, Southern Medical University, Guangzhou, 510282 China; 2 Department of Environmental Health, School of Public Health, Southern Medical University, Guangzhou, 510515 China; 3 Guangdong Provincial Institute of Public Health, Guangdong Provincial Center for Disease Control and Prevention, Guangzhou, 511430 China; 4 Department of Pathophysiology, Southern Medical University, Guangzhou, 510515 China; 5 Shenzhen Fun-Poo Biotech Co., Ltd., Shenzhen, 518000 China; 6 Department of Pathogen Biology, School of Public Health, Southern Medical University, Guangzhou, 510515 China; 7 Guangdong Provincial Center for Disease Control and Prevention, Guangzhou, 511430 China; 8 Department of Neurology, NanFang Hospital, Southern Medical University, Guangzhou, 510515 China; 9 Department of Microbiology and Immunology, KU Leuven–University of Leuven, Leuven, Belgium; 10 VIB, Center for the Biology of Disease, Leuven, Belgium; 11 Vrije Universiteit Brussel, Faculty of Sciences and Bioengineering Sciences, Microbiology Unit, Brussels, Belgium

**Keywords:** Metabolic syndrome, Faecal microbiome, Guangdong Gut Microbiome Project, Epidemiology, Population level survey, Economic status, 16S rRNA gene sequencing

## Abstract

**Background:**

The metabolic syndrome (MetS) epidemic is associated with economic development, lifestyle transition and dysbiosis of gut microbiota, but these associations are rarely studied at the population scale. Here, we utilised the Guangdong Gut Microbiome Project (GGMP), the largest Eastern population-based gut microbiome dataset covering individuals with different economic statuses, to investigate the relationships between the gut microbiome and host physiology, diet, geography, physical activity and socioeconomic status.

**Results:**

At the population level, 529 OTUs were significantly associated with MetS. OTUs from Proteobacteria and Firmicutes (other than Ruminococcaceae) were mainly positively associated with MetS, whereas those from Bacteroidetes and Ruminococcaceae were negatively associated with MetS. Two hundred fourteen OTUs were significantly associated with host economic status (140 positive and 74 negative associations), and 157 of these OTUs were also MetS associated. A microbial MetS index was formulated to represent the overall gut dysbiosis of MetS. The values of this index were significantly higher in MetS subjects regardless of their economic status or geographical location. The index values did not increase with increasing personal economic status, although the prevalence of MetS was significantly higher in people of higher economic status. With increased economic status, the study population tended to consume more fruits and vegetables and fewer grains, whereas meat consumption was unchanged. Sedentary time was significantly and positively associated with higher economic status. The MetS index showed an additive effect with sedentary lifestyle, as the prevalence of MetS in individuals with high MetS index values and unhealthy lifestyles was significantly higher than that in the rest of the population.

**Conclusions:**

The gut microbiome is associated with MetS and economic status. A prolonged sedentary lifestyle, rather than Westernised dietary patterns, was the most notable lifestyle change in our Eastern population along with economic development. Moreover, gut dysbiosis and a Western lifestyle had an additive effect on increasing MetS prevalence.

**Electronic supplementary material:**

The online version of this article (10.1186/s40168-018-0557-6) contains supplementary material, which is available to authorized users.

## Background

Dysbiosis in the human gut microbiota causes or aggravates metabolic disorders by affecting host energy balance, chronic inflammation and feeding behaviour [1–4]. In addition, the gut microbiota of Western populations harbours higher obesogenic capabilities than the gut microbiota of populations from under-developed nations [5]. These findings imply relationships among economic development, gut microbiota shifts and the metabolic syndrome (MetS) epidemic. The morbidity of metabolic diseases is increasing rapidly in Eastern nations, likely due to the drastic lifestyle transitions that occur during economic development and urbanisation [6–8]. Due to the unbalanced economic development across Eastern nations, these areas provide opportunities to observe the associations among economic status, gut microbiota and metabolic diseases.

We recently performed the largest Eastern-nation gut microbiome epidemiological survey, which included 7009 individuals in South China, using a multi-stage cluster sampling scheme (Guangdong Gut Microbiome Project, GGMP) [9]. Our preliminary observations were that the human gut microbiota exhibits large regional variations but that it is possible to identify consistent gut microbial signatures for metabolic syndrome by performing population-level analyses within a large region. In the present study, we utilised the GGMP dataset to reveal the gut microbiome signatures of MetS and aimed to analyse the relationships among gut dysbiosis, host economic status and the metabolic disease epidemic.

## Results

### The overall gut microbiome configuration is correlated with MetS

We previously described the population included in the GGMP (Guangdong Gut Microbiome Project) [9]. A total of 6896 individuals were included in the present analysis according to our inclusion criteria (Additional file 1: Supplementary Methods, lines 25–27), of which 1404 (20.4%) were diagnosed with MetS (Table 1). High systolic blood pressure (SBP) was the most common disorder found in the population, followed by low high-density lipoprotein (HDL). There was approximately equal representation of the two genders in the population. MetS and related disorders were significantly more prevalent in men than in women.

**Table 1:** Characteristics of the study participants

	Female (*n* = 3803)	Male (*n* = 3093)	*P* value
Age (years, mean ± SD)	51.9 ± 14.4	53.7 ± 14.9	< 0.001
Waist ≥ 90 cm (male) or ≥ 85 cm (female), *n* (%)	963 (25.3)	678 (21.9)	0.001
BP ≥ 130/85 mmHg, *n* (%)	1775 (46.7)	1636 (52.9)	< 0.001
SBP ≥ 130 mmHg, *n* (%)	1709 (44.9)	1557 (50.3)	< 0.001
DBP ≥ 85 mmHg, *n* (%)	773 (20.3)	916 (29.6)	< 0.001
TG ≥ 1.7 mmol/L, *n* (%)	728 (19.1)	836 (27.0)	< 0.001
HDL < 1.04 mmol/L, *n* (%)	799 (21.0)	1107 (35.8)	< 0.001
FBG ≥ 6.1 mmol/L, *n* (%)	628 (16.5)	584 (18.9)	0.011
MetS, *n* (%)	657 (17.3)	747 (24.2)	< 0.001

Significant differences were determined by a *t* test for age and chi-square tests for the remaining factors

**Fig. 1:**
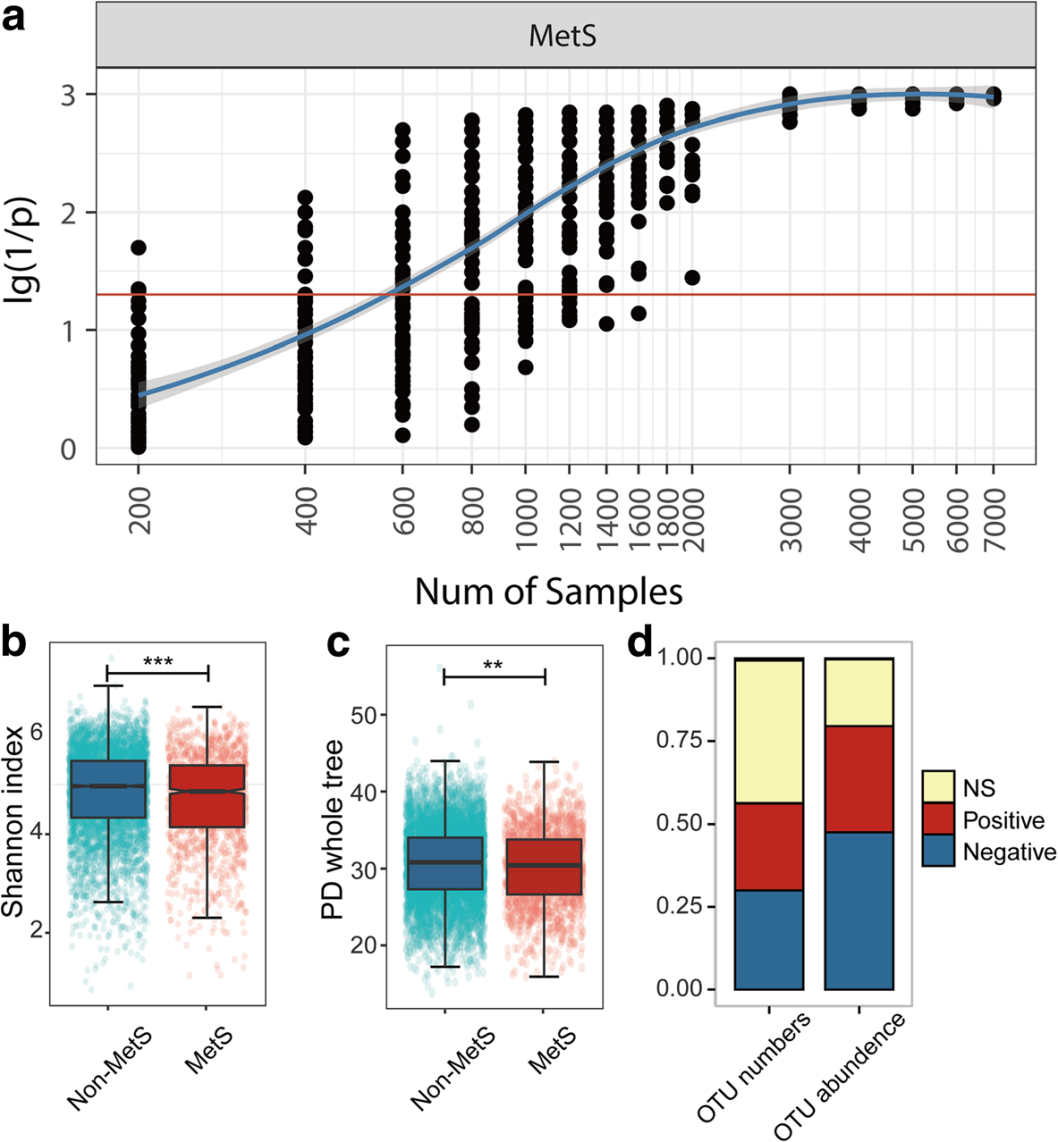
The overall gut microbial community is associated with MetS. **a** Significance, represented by log_10_ transformed (1/*p*) value, of ADONIS test associating gut microbiota variations and MetS at different sample sizes, with 50 replicates at each step. The red line indicates *p* = 0.05. **b** Shannon indices comparing MetS subjects (MetS, *N* = 1404) with the remainder of the population (non-MetS, *N* = 5492). **c** PD whole tree indices comparing MetS subjects (MetS, *N* = 1404) with the remainder of the population (non-MetS, *N* = 5492). Wilcoxon rank-sum test adjusted by the Benjamini and Hochberg method (**b**, **c**). ***P < 0.001, **P < 0.01. **d** Proportions of OTUs associated with MetS in terms of OTU number and accumulated abundance

Through QIIME analyses [10], a total of 17,083 16S ribosomal small subunit 97% OTUs were obtained after the samples were rarefied to 10,000 sequences. To determine whether our sampling size was adequate to detect significant correlations between the gut microbiome and MetS, we examined significance using Adonis analysis with various subsample sizes and 50 replications per size. As illustrated by the Adonis *p* values, larger sample sizes resulted in lower *p* values (or higher 1/*p*, Fig. 1a), and it was determined that approximately 1800 samples were required to confidently obtain significant correlations between MetS and the gut microbiome for all 50 replications at this sample size. This finding indicates that our sample size was sufficient for identifying microbial signatures for metabolic disorders in the population. As demonstrated by the Shannon and phylogenetic diversity (PD) whole tree indices, microbial diversities were significantly lower in MetS subjects than in the remainder of the population (Fig. 1b, c).

To identify microbiome signatures of MetS, we performed MaAsLin (multivariate association with linear models) to associate host metadata with gut microbial species [11, 12]. MaAsLin performs boosted, additive general linear models between metadata (predictors) and microbial species’ abundances (response variables), and reports beta values of modelling (coefficients) and statistical significance after controlling for the false discovery rate (*q* value). MaAsLin can detect the effect of one metadata category while de-confounding the effects of others. By implementing MaAsLin, we examined the associations between OTUs and MetS status and between OTUs and the condition’s diagnostic factors in a stringent manner (Additional file 1: Supplementary Methods, line 49 to 65). We also discarded OTUs that existed in less than 10% of our participants from this analysis, with 930 OTUs remaining for further analysis. Five hundred twenty-nine of the 930 analysed OTUs, constituting approximately 80% of the total abundance, were significantly associated with MetS or its related factors (Fig. 1d). Among the 1243 associations between OTUs and MetS or its diagnostic factors, 676 were negative associations, and 567 were positive associations. The associations with OTUs were as follows: waist (184− negative and 175+ positive associations, represented as “184−” and “175+” hereafter), triglycerides (TG, 149− and 153+), systolic blood pressure (SBP, 92− and 43+), diastolic blood pressure (DBP, 72− and 29+), fasting blood glucose (FBG, 21− and 40+), HDL (34+ and 16−) and MetS status (124− and 111+). The full list of associations is provided in Additional file 2: Table S1.

### Association patterns between gut species and MetS were phylogenetically consistent

**Fig. 2:**
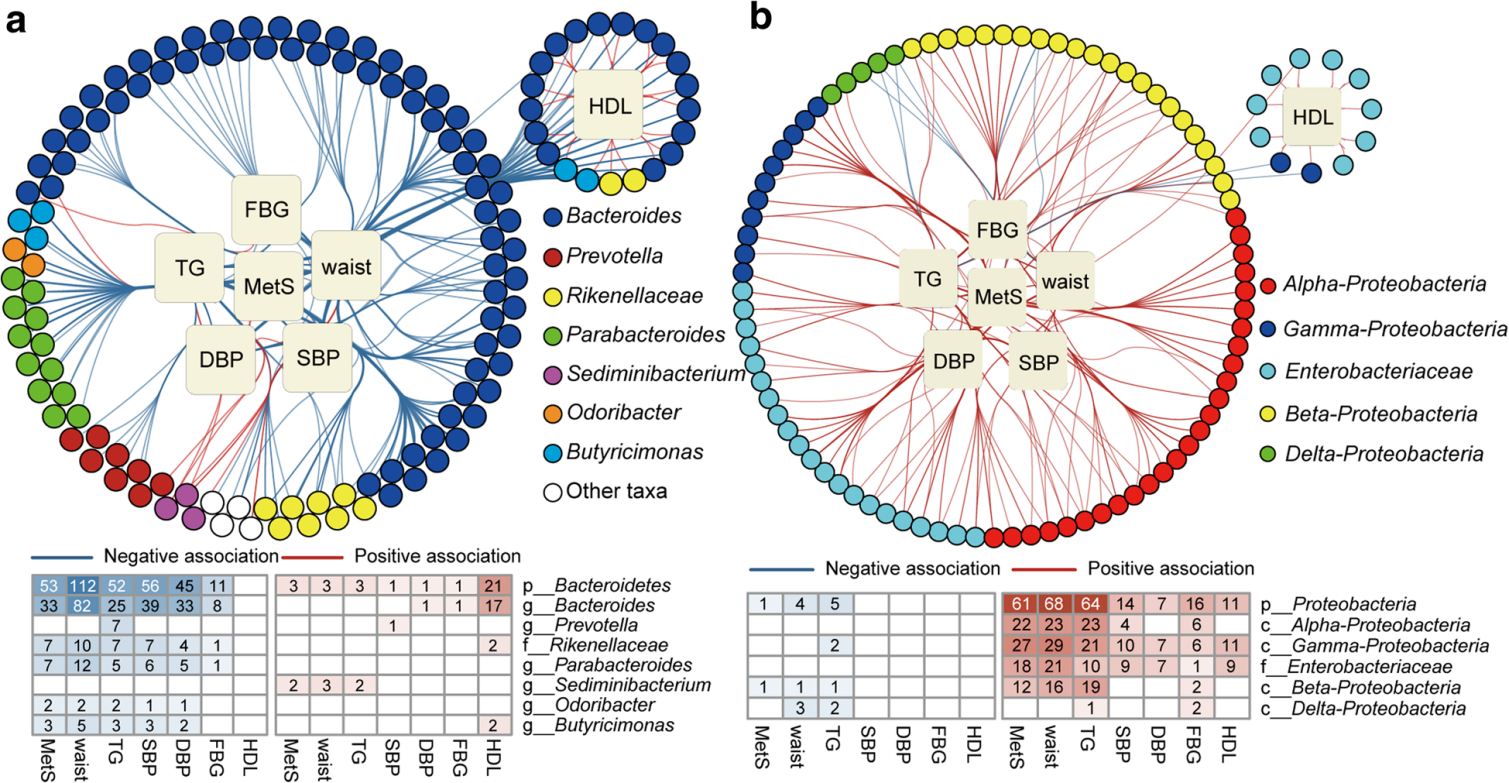
Associations between MetS and OTUs from Bacteroidetes and Proteobacteria. Network showing significant associations between (**a**) Bacteroidetes and (**b**) Proteobacteria OTUs and MetS. Squares represent metadata, and circles represent OTUs, which are connected with red or blue edges where significantly positively or negatively associated, respectively. Edges are bundled for clearer visualisation. The actual number of associations is summarised below the network

In our data, Bacteroidetes, Proteobacteria and Firmicutes contributed 92.3% of the total abundance and the majority of the associations with MetS. For Bacteroidetes, 129 of its OTUs were associated with MetS, constituting approximately 79.2% of the total abundance of Bacteroidetes and 27.9% of the entire community (Fig. 2a, Additional file 1: Figure S1a). These 129 OTUs contributed to 362 associations with MetS and its diagnostic factors, and almost all of these associations were negative (350− and 12+, HDL was rescored with the opposite valence when added to other factors here and in the following results). For Proteobacteria, 96 of its 137 OTUs were associated with MetS or its diagnostic factors and constituted 93.4% of the total Proteobacteria abundance and 15.3% of the entire community (Fig. 2b, Additional file 1: Figure S1b). The 137 Proteobacteria OTUs exhibited 251 associations with MetS and its diagnostic factors, and almost all of these associations were positive (230+ and 21−).

**Fig. 3:**
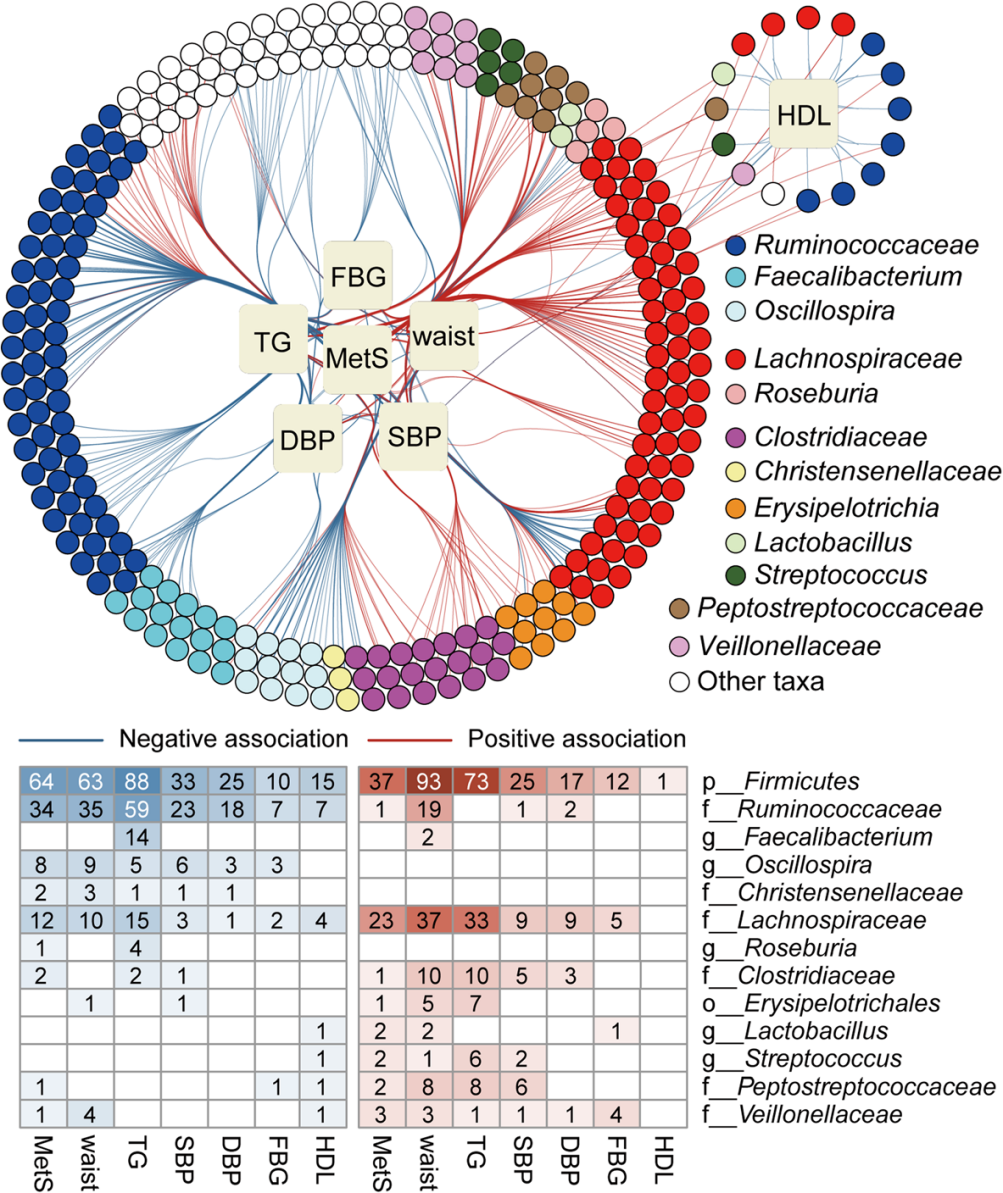
Associations between MetS and OTUs from Firmicutes. Network showing the significant associations between Firmicutes OTUs and MetS. The figure structure is similar to that of Fig. 2

The roles of the Firmicutes phylum in metabolic diseases are known to be complex, and previous reports have been inconsistent [13–15]. Such inconsistency was also captured in our population (Fig. 3, Additional file 1: Figure S1c), as 272 positive associations and 284 negative associations were observed between Firmicutes OTUs and MetS or its diagnostic factors. Within this phylum, phylogenetic conservativeness was observed at the family level for the associations between gut species and MetS. OTUs from Ruminococcaceae (176− and 30+) and Christensenellaceae (8− and 0+) tended to associate negatively with MetS, whereas OTUs from other Firmicutes, including Lachnospiraceae (43− and 120+), Clostridiaceae (5− and 29+), Veillonellaceae (5− and 14+), and Peptostreptococcaceae (2− and 25+), tended to associate positively with MetS. The association patterns persisted when using other clustering methods or confounders (Additional file 1: Supplementary Results, line 202 to 261).

In addition to the above dominant phyla, OTUs from other phyla, including Actinobacteria (0− and 35+), Fusobacteria (1− and 16+, all with *Fusobacterium*), Verrucomicrobia (7− and 0+, all with *Akkermansia*), Tenericutes (9− and 0+, all with RF39), Synergistetes (1− and 0+, all with *Synergistes*) and Euryarchaeota (3− and 0+, all with *Methanobrevibacter*), were also correlated with MetS or its diagnostic factors (Additional file 1: Figure S2). The Actinobacteria OTUs were highly diverse, with associations involving two classes and seven families, which were all positively associated with MetS or related factors (Additional file 2: Table S1). *Bifidobacterium* OTUs were positively associated with FBG, and these associations persisted after adjusting for the consumption of pre-/pro-/sym-biotics (Additional file 2: Table S2). More detailed descriptions of associations between OTUs and MetS are provided in Additional file 1: Supplementary Results, lines 104 to 184.

### Associations between gut microbiota, economic status and metabolic syndrome

**Fig. 4:**
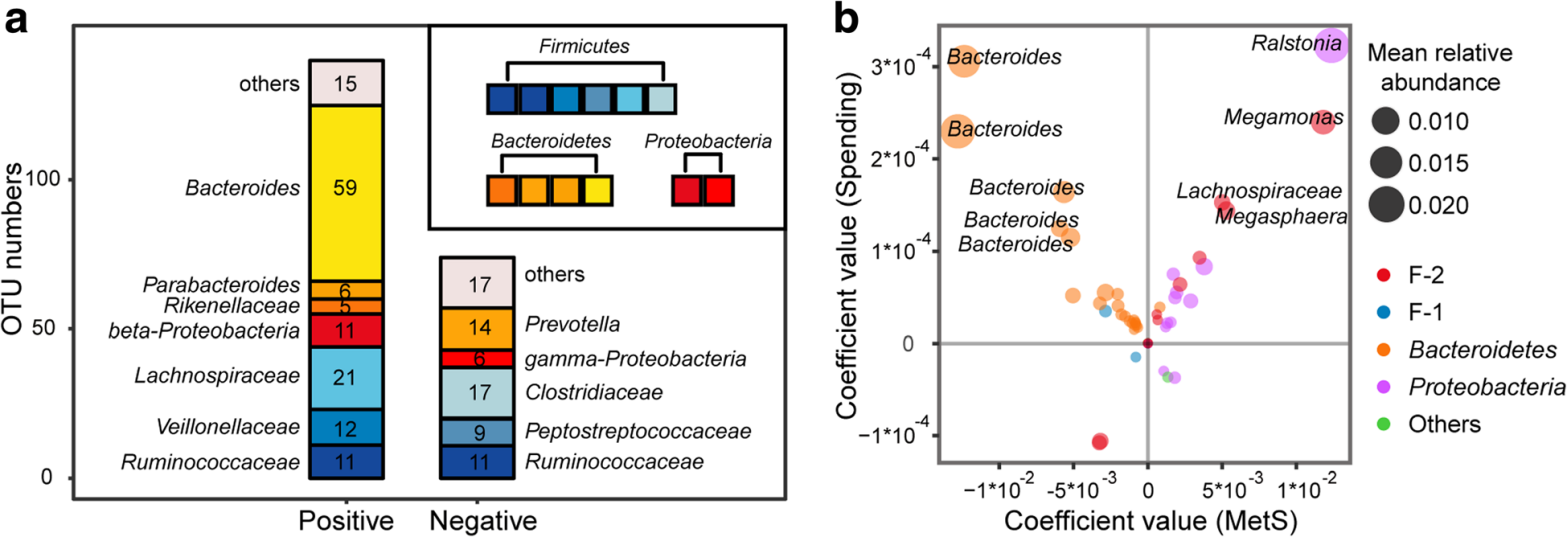
Associations between OTUs and host economic status. **a** Stacked plot showing the number of OTUs that are positively or negatively associated with income or spending. Colours correspond to taxonomies in the legend. **b** Four-quadrant diagram showing the coefficients of OTUs with MetS (*x*-axis) and spending (*y*-axis). OTUs that are significantly associated with MetS and economic status simultaneously were plotted

According to data from the Statistics Bureau of Guangdong Province, in 2015, our sampling regions covered both underdeveloped cities, such as Meizhou and Wuhua (Gross Domestic Product (GDP) per capita, 22.1 k and 25.3 k CNY, respectively), and developed ones, such as Shenzhen and Guangzhou (GDP per capita, 149.5 k and 136.1 k CNY, respectively). We collected data on participant yearly income and spending by questionnaire to represent participant economic status. The median income and spending in each sampling area were well correlated with local GDP per capita, indicating that self-reported income and spending reliably reflected participant economic status (Additional file 1: Figure S3). Using gender, age and Bristol stool scale as confounders, we found a total of 214 OTUs, representing 38.3% of the total microbial abundance, that were significantly associated with personal income or spending (Additional file 2: Table S3). Among these OTUs, 140 were positively correlated with economic status, comprising 59 *Bacteroides* OTUs, 6 *Parabacteroides* OTUs, 5 Rikenellaceae OTUs, 21 Lachnospiraceae OTUs, 11 Ruminococcaceae OTUs, 12 Veillonellaceae OTUs and 11 beta-Proteobacteria OTUs, whereas 74 OTUs were negatively associated with individual economic status, comprising 14 *Prevotella* OTUs, 17 Clostridiaceae OTUs, 9 Peptostreptococcaceae OTUs, 11 Ruminococcaceae OTUs and 4 Enterobacteriaceae OTUs (Fig. 4a). Among these 214 OTUs associated with economic status, 157 were also significantly associated with MetS or its diagnostic factors. However, we did not find a general consistency between correlations of OTUs with MetS and those of OTUs with economic status in our sampled population, as OTUs that were positively correlated with economic status were either positively or negatively correlated with MetS (Fig. 4b). For example, of the four OTUs that showed the highest positive correlation coefficients with economic status, two of them, which were two *Bacteroides* OTUs, were negatively correlated with MetS, whereas the other two, one *Ralstonia* OTU and one *Megamonas* OTU, were positively correlated with MetS.

**Fig. 5:**
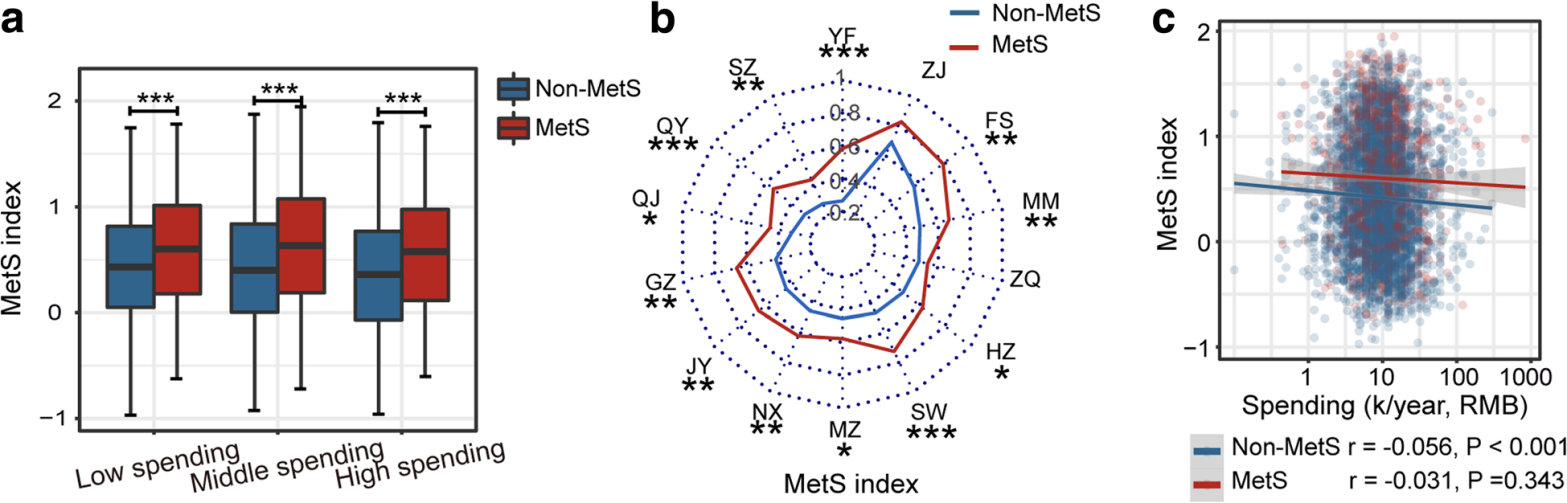
Validating the MetS index and its association with economic status. **a**, **b** The MetS index compared between MetS subjects and non-MetS subjects in individuals with different economic status using boxplots (**a**) and in individuals from different sampling regions using a radar chart (**b**). In (**b**), the median MetS index values of MetS and non-MetS subjects in each region were plotted along with radar angles, and each angle represents one sampled region. Wilcoxon rank-sum test adjusted by the Benjamini and Hochberg method (**c**, **d**). ***P < 0.001, **P < 0.01, *P < 0.05. **c** Correlations between the MetS index and spending in MetS and non-MetS subjects evaluated by Spearman correlation tests

To explore the correlation of MetS-associated gut dysbiosis with economic status, we formulated a gut microbial MetS index to represent the overall gut dysbiosis associated with MetS for a given sample (Additional file 1: Supplementary Methods, lines 77 to 82). This index was the accumulated abundance of positively and negatively MetS-associated OTUs weighted by their coefficient and significance values. To determine whether this index could reliably reflect gut dysbiosis, we investigated whether the MetS index was consistently higher in MetS subjects than in non-MetS subjects in different subpopulations. Our results showed that regardless of economic status or sampling city, the MetS indices were higher in MetS subjects than the other subjects, suggesting the applicability of this index (Fig. 5a, b). Interestingly, rather than observing a positive correlation between gut microbial MetS index and host economic status, we observed a slight but significant negative correlation between these two parameters in non-MetS subjects (*r* = − 0.056, *p* < 0.001) (Fig. 5c), indicating that, in our population, the gut microbiota of people of higher economic status did not show more signs of MetS in their gut microbiome compositions than that of lower economic status individuals.

### Sedentary lifestyle in combination with gut microbiota dysbiosis showed an additive effect in increasing metabolic syndrome prevalence

**Fig. 6:**
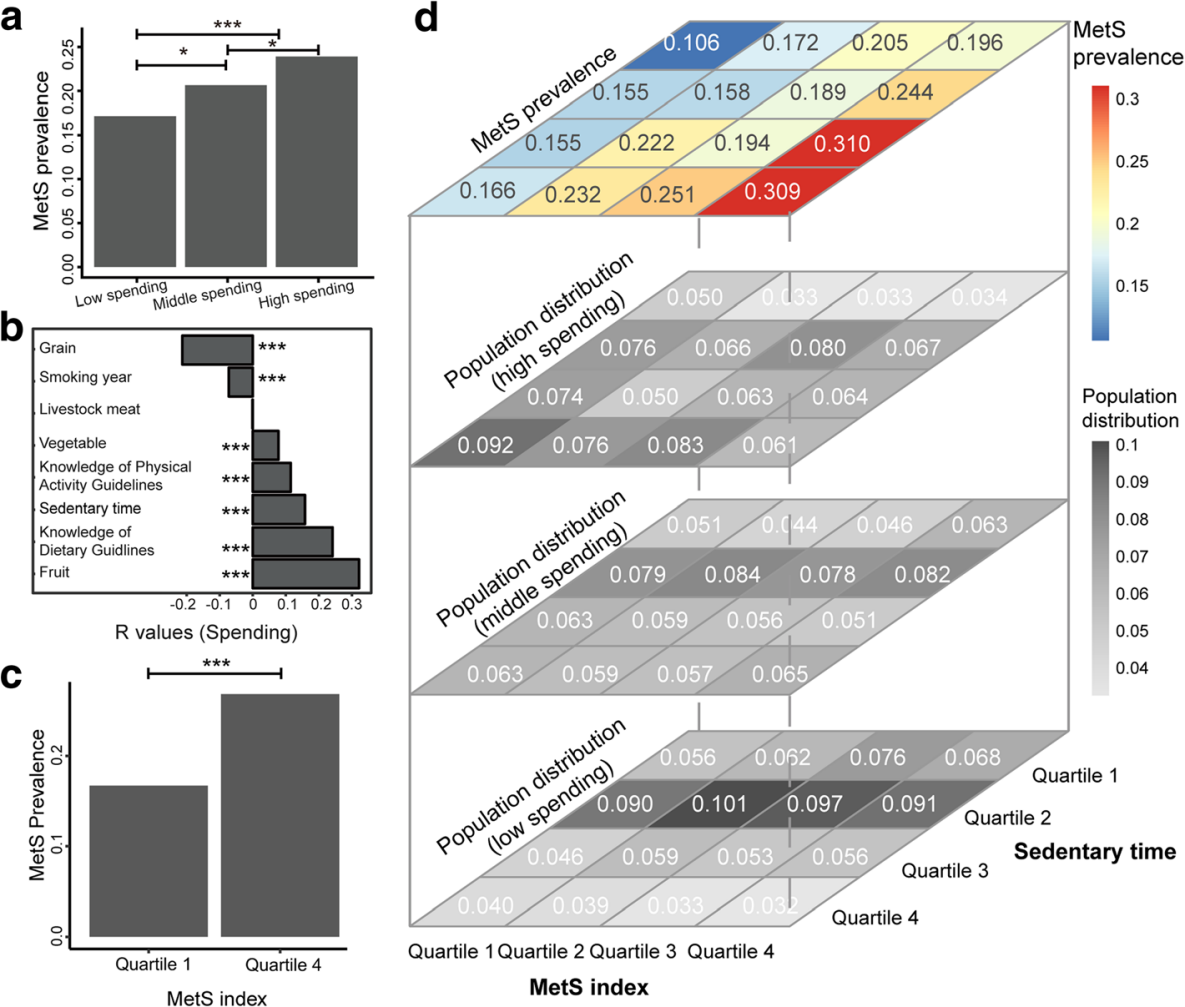
Relationships among host economic status, MetS prevalence and lifestyle. **a** MetS prevalence in individuals of differing economic status. Individuals were classified into low-spending (*N* = 1170), moderate-spending (*N* = 2394) and high-spending (*N* = 1099) groups, and the ratios of MetS subjects of each group were compared by a chi-square test, with adjustment by the Benjamini and Hochberg method; ***P < 0.001, *P < 0.05. **b** Bar plot illustrating correlation coefficient values for host spending and lifestyle. A longer bar indicates a higher coefficient, and the *R* values are labelled on the *x*-axis. Correlation coefficients were calculated by Spearman correlation, and the Benjamini and Hochberg method was used to adjust for multiple comparisons. ***P < 0.001. **c** Comparison of MetS prevalence between subjects with low MetS index values (*N* = 1719) and high MetS index values (*N* = 1714), with analysis performed via a chi-square test. **d** MetS prevalence in individuals with different MetS index values. Individuals were quarterised according to their MetS index values, and the ratio of MetS subjects in each quartile was compared by a chi-square test. The top 16-grid plot shows the MetS prevalence in different subpopulations. Individuals were divided into 16 groups according to the quarterisation of their MetS index (*x*-axis) and sedentary time values (*y*-axis). The colour gradient of the cell indicates MetS prevalence, which is also indicated in each cell. The results of the statistical tests between all pair of cells are provided in Additional file 2: Table S4. The three bottom 16-grid plots show population distributions within each spending level. Individuals were first divided into high-(the second plot), moderate-(the third plot) and low-spending (the fourth plot) groups, and the proportion of individuals in each cell at their economic level was calculated. The greyscale of the cell indicates the proportion, which is also indicated in each cell

The prevalence of MetS usually increases with economic development. This was observed in our population. MetS prevalence was significantly higher in individuals with higher economic status (26.8%) than in those of with lower economic status (16.7%) (Fig. 6a). To understand the reasons behind this phenomenon, we explored the correlations of personal economic status with diet habits as well as with lifestyle habits. With increasing economic status, diet patterns did not become increasingly Westernised in our investigated population. The amount of meat consumption did not vary with economic status, whereas vegetable and fruit consumption significantly increased and grain consumption decreased with increasing economic status (Fig. 6b). In addition, we observed that people of higher economic status in our population had more awareness of public health knowledge, which might partially explain the different dietary patterns. Accordingly, smoking was also reduced at higher economic status. Nevertheless, sedentary lifestyle significantly increased with increasing personal spending.

We wondered if MetS-associated gut dysbiosis had an additive effect with increased sedentary time on MetS prevalence. To investigate this possibility, we quarterised our participants according to MetS index and calculated the MetS prevalence in each quartile. The results showed that MetS was significantly more prevalent in the 4th quartile (26.3%) than in the 1st quartile (14.7%) (Fig. 6c). Furthermore, when we added sedentary time as a synergistic factor and re-classified our population, the discrepancy in MetS prevalence among quartiles was further enlarged, with 30.9% of individuals with both long-term sedentary lifestyle and high microbial MetS index suffering from MetS. This percentage was almost triple the percentage of individuals with MetS within the 1st quartile for both MetS index and sedentary time (10.6%). The percentages of people with MetS who had a high MetS index but low sedentary lifestyle (19.6%) or a low MetS index but a high sedentary lifestyle (16.6%, Fig. 6d and Additional file 2: Table S4) were between the two. With increasing economic status, we observed a shift from fairly active (low sedentary time) to sedentary lifestyles and, consequently, a shift from lower to higher risk patterns (Fig. 6d). Accordingly, the additive effect of sedentary time and gut microbiome dysbiosis contributed to the increased rate of MetS in people of higher economic status.

## Discussion

The present study used a dataset, the Guangdong Gut Microbiome Project (GGMP), with a stringent protocol applied for the data collection process [9]. We utilised a stratified, multi-level sampling scheme, with nearly 7000 subjects included in the final analyses. The identification of MetS individuals was based on blood tests performed and measured anthropometric values collected in situ by three trained faculty members. To adjust the multivariable association analysis, age, gender, geographic location and the Bristol stool scale were included to account for confounding effects [16–20], and a stringent false discovery rate (FDR) threshold was applied to minimise false discoveries. These processes allowed us to identify the microbiome signatures associated with metabolic diseases with high confidence. More than half of the analysed OTUs, constituting up to 80% of the total abundance and spanning almost all phyla, were significantly associated with metabolic disorders. Although many studies have reported correlations between gut microbiota and metabolic diseases [21–24], this high proportion shows that gut microbes are generally correlated with the metabolic status of the host.

Our results for the large study population show that similar taxonomic groups generally yield similar results with respect to their positive or negative correlations with metabolic disorders. It is possible that OTUs from phyla with low levels of diversity, e.g. Verrucomicrobia (dominated by *Akkermansia*), Fusobacteria (dominated by *Fusobacterium*) and Tenericutes (dominated by RF39), may have similar correlation directions with MetS. Nevertheless, OTUs from major taxa with very high levels of diversity, such as Bacteroidetes, Proteobacteria, Actinobacteria and Ruminococcaceae, showed similar relationships within each taxon to metabolic disorders. These similar patterns indicate that, although the phylogenetic classification of microbes is not necessarily related to their functions, many phylogenetic groups contain members with similar traits regarding their relationships with host metabolism [25].

The relationships between some gut microbial taxa and host metabolism reported in Western populations were also recaptured in our population. For example, *Akkermansia* was found to be an important metabolism-regulating bacterium in recent clinical trials [26, 27]. In our population, an *Akkermansia* OTU showed the highest negative coefficient with MetS, indicating that this bacterium is among the most important beneficial bacteria for host metabolism in our population. The similar gut microbial taxa alterations between Eastern and Western populations in metabolic disorders also included OTUs from Firmicutes (e.g. *Blautia*, *Clostridium*, *Megasphaera*, *Roseburia*, *Faecalibacterium*, Christensenellaceae and *Oscillospira*) and Proteobacteria [28–32]. Such findings suggest that, even with large variations in ethnicity, lifestyle, dietary habits, socioeconomic status and environmental factors between Western and Eastern populations, gut microbial dysbiosis patterns for MetS could be similar between Western and Eastern nations. These findings provide supporting justification for efforts to identify universal microbiota targets for metabolic diseases [33], even if microbiota-based disease models might not be equally applicable among different locations [9], to facilitate the delivery of precise nutrition and medicine in the future [34].

The present study provides additional novel and interesting insights regarding the relationships between gut microbes and metabolic diseases. We found that *Fusobacterium*, an important colonic carcinogenic bacterium [35], was positively correlated with metabolic disorders, indicating that this bacterium might participate in both colon cancer and metabolic diseases. *Lactobacillus* was positively associated with metabolic disorders after adjusting for the consumption of pre-/pro-/sym-biotics, consistent with previous reports that this bacterium is enriched in obese patients [36] and in patients with type 2 diabetes [23], MetS [37], stroke [38] and rheumatoid arthritis [39]. Similarly, we found that *Bifidobacterium* OTUs were positively associated with FBG. This finding seems to conflict with conventional understanding, as *Lactobacillus* and *Bifidobacterium* are two major groups used in probiotics but are enriched in metabolic disorders. Our cross-sectional study does not allow us to argue against the reported benefits of these traditional probiotics, as causality cannot be inferred based on the data reported here, and the apparent contradiction might result from strain-level specificity in bacterial functions. Nevertheless, the roles of these two genera in host metabolism under nutrient-rich conditions should be investigated more thoroughly.

The present study provided evidence that gut microbes are associated with economic status. In our study, more than 200 OTUs, constituting approximately 38.3% of the total gut microbial abundance, were correlated with individual yearly income or spending, indicating general associations between gut microbes and host economic status. OTUs from gamma-Proteobacteria were negatively associated with economic status, which agrees with the observation that this taxon is prevalent in less-developed areas [17, 33, 40]. Interestingly, in an analysis of the gut microbiome over a 1-year period of a subject who travelled to developing nations in Southeast Asia, a sharp increase in gamma-Proteobacteria was found [41], supporting the idea that environments in less-developed areas may produce an enrichment of species from gamma-Proteobacteria in the guts of those living there. In our study, OTUs from *Bacteroides* were positively correlated with host economic status, whereas those from *Prevotella* were negatively correlated with host economic status. It was previously reported that children from Europe harbour a higher proportion of gut *Bacteroides*, whereas those from rural Africa harbour a higher proportion of gut *Prevotella* [5]. Our observations indicate that such a trend might be related to decreased grain consumption in individuals of higher economic status [42].

OTUs that were associated with economic status were generally also associated with MetS or its diagnostic factors. Nevertheless, the gut microbiota did not consistently shift to a more dysbiotic status with economic development. In our population, meat consumption was not significantly and positively associated with economic status, whereas vegetable and fruit consumption were. These findings might be because livestock meat is not a limited food resource in Guangdong Province, which is one of the wealthiest regions in China. (The GDP of Guangdong Province ranked 1st in China according to data from National Bureau of Statistics of China in 2015.) Furthermore, individuals of higher economic status were more aware than were those of lower economic status of relevant public health knowledge and were thus more likely to develop a healthier diet.

The potential addictive effect of gut dysbiosis and lifestyle is an interesting finding. Our results only superficially addressed this relationship, and further studies are needed to establish causality. Fei et al. isolated a bacterial strain, *Enterobacter cloacae B29*, from an obese man, that induced obesity and insulin resistance in germ-free mice fed a high-fat diet but not in those fed a normal chow [43], suggesting that diet may have additive effects with gut dysbiosis as well. The observed additive effect of gut dysbiosis and lifestyle implies that the risk of metabolic diseases from gut dysbiosis is high in underdeveloped areas if people transition to a Western lifestyle. We suggest that the co-occurrence of gut dysbiosis and lifestyle change in developing areas may play additive roles in metabolic diseases and that additional studies on this topic are warranted with additional cofactors, such as genetics, diet and their interactions.

## Conclusion

The present analysis, based on a sample size of nearly 7000 participants in an Eastern nation, revealed gut microbiota alterations associated with MetS. These alterations were phylogenetically conserved and showed similarities to those reported in Western populations. Furthermore, our study indicated a relationship between the gut microbiome and economic development and provided preliminary evidence that gut dysbiosis interacts with Western lifestyle habits to affect MetS prevalence. We propose that the gut microbiome may provide potential targets for epidemic metabolic diseases.

## Methods

### Description of the dataset

We previously described the Guangdong Gut Microbiome Project (GGMP) [9]. Briefly, the project was conducted in South China across 14 preselected districts/counties corresponding to various degrees of economic development. Three neighbourhoods/townships were randomly selected in each district/county, and two communities/villages were randomly selected in each neighbourhood/township using probability proportional to size (PPS) sampling. Approximately 7000 subjects were investigated, and their gut microbiomes were profiled by sequencing the V4 region of the 16S rRNA gene.

### Bioinformatics and biostatistics

Processing of the raw Illumina sequences was mainly based on the Greengenes database [44] of QIIME (1.9.1) software [10] and was identical to that described in our previous reports [9]. We performed multivariate association analyses with linear modelling (MaAsLin) as described by Morgan et al. [12] to examine associations between prevalent operational taxonomic units (OTUs, detected in at least 10% of all samples) and each of MetS status and several diagnostic factors. Age, gender, geographic location and the Bristol stool scale were used as confounders, and the false discovery rate was limited to 0.05. R (3.2.2) statistical software was used for data plotting and statistical analyses. The significance of differences between two groups was determined by the Wilcoxon rank-sum test. The Spearman’s rank correlation test was used to analyse the correlation between two variables. The chi-square test was used to compare the ratios of two groups. *P* values less than or equal to 0.05 were considered significant. The Benjamini and Hochberg method was used to adjust the *P* value for multiple hypotheses.

Detailed descriptions can be found in Additional file 1: Supplementary Methods and Materials.

## Additional files

Additional file 1: Supplementary Data: contains Supplementary Methods and Materials, Supplementary Results, Supplementary Figures and Legends, References for Supplementary Data. (DOCX 3627 kb)

Additional file 2: Supplementary Tables S1-S13. (XLSX 1996 kb)

## Abbreviations

BP: Blood pressure
DBP: Diastolic blood pressure
FBG: Fasting blood glucose
FDR: False discovery rate
GDP: Gross domestic product
GGMP: Guangdong Gut Microbiome Project
HDL: High-density lipoprotein
MaAsLin: Multivariate analysis by linear models
MetS: Metabolic syndrome
OTUs: Operational taxonomic units
PD whole tree: Phylogenetic diversity whole tree
SBP: Systolic blood pressure
TG: Triglyceride
