# Supplementary material for: Linking gut microbiota, metabolic syndrome and economic status based on a population-level analysis
Source: Microbiome. 2018 Sep 24;6:172. doi: 10.1186/s40168-018-0557-6 (PMC6154942; doi:10.1186/s40168-018-0557-6)
Supplement: Supplementary file 1 — Supplementary Data: contains Supplementary Methods and Materials, Supplementary Results, Supplementary Figures and Legends, References for Supplementary Data. (DOCX 3627 kb) [file 40168_2018_557_MOESM1_ESM.docx]

**Supplementary Data**

Linking gut microbiota, metabolic syndrome and economic status based on a population-level analysis

Yan He^1#^, Wei Wu^2,3^^#^, Shan Wu^1,2#^, Hui-Min Zheng^1,2#^, Pan Li^1,2#^, Hua-Fang Sheng^1^, Mu-Xuan Chen^1^, Zi-Hui Chen^3^, Gui-Yuan Ji^3^, Zhong-Dai-Xi Zheng^2^, Prabhakar Mujagond^1^, Xiao-Jiao Chen^1^, Zu-Hua Rong^1,2^, Peng Chen^4^, Li-Yi Lyu^5^, Xian Wang^5^, Jia-Bao Xu^6^, Chong-Bin Wu^5^, Nan Yu^1^, Yan-Jun Xu^7^, Jia Yin^8^, Jeroen Raes^9,10,11^, Wen-Jun Ma^3*^, Hong-Wei Zhou^1*^

**This supplementary data file contains the following:**

Supplementary Methods and Materials

Supplementary Results

Supplementary Figures and Legends

References for Supplementary Data

**SUPPLEMENTARY METHODS AND MATERIALS**

**Metadata collection and metabolic syndrome determination**

The data used in the present analysis were from the Guangdong Gut Microbiome Project and were described previously(1). Metabolic syndrome (MetS) was determined according to the Joint Committee for Developing Chinese Guidelines on Prevention and Treatment of Dyslipidemia in Adults based on a subject's meeting of three of the five following criteria: (1) waist>90 cm (male) or waist>85 cm (female), (2) FBG≥6.1 mmol/L (110 mg/dl) or previous diagnosis of diabetes mellitus, (3) TG≥1.7 mmol/L (150 mg/dl), (4) HDL<1.04 mmol/L (40 mg/dl), and (5) SBP/DBP≥130/85 mmHg or previous diagnosis of high blood pressure. Subjects with inadequate metadata for MetS diagnosis were excluded from the present analysis, and a total of 6879 subjects were retained (Additional file 2: Table S5).

The Guangdong Gut Microbiome Project used in-person questionnaire interviews to collect subject metadata (Additional file 2: Table S5). In the present analysis, we used age and gender as confounders in the multivariate association analysis. To evaluate how medication and non-metabolic syndrome disease status might affect the associations between metabolic disorders and gut microbes, we also compiled information on usage of antibiotics and pre-/pro-/sym-biotics; whether individuals with hypertension, diabetes or dyslipidaemia were taking medicine to control their corresponding symptoms; and whether the subjects had IBD, IBS, colorectal carcinoma or rheumatoid arthritis. We also added participant annual income and spending for each investigated household to analyse correlations between gut microbiota and personal economic status in the present analysis. We also asked the volunteers to estimate their lifestyle parameters to study the relationships of these parameters with economic status and gut microbiome.

**Bioinformatics and biostatistics**

Pre-processing of the raw sequences was described previously(1). We used the Shannon index and PD whole tree to represent alpha diversity. A higher Shannon index indicates a higher number of microbial species in a sample and a more even distribution of microbial species. A higher PD whole tree value indicates a wider range of phylogenetic distances among microbial species. Both indices were calculated using QIIME, and comparisons between MetS subjects and the rest of the population were performed by Wilcoxon rank sum test.

To identify microbiome signatures of MetS, we performed multivariate association analyses (MaAsLin) to examine the associations between OTUs and both MetS status and its diagnostic factors(2, 3). We performed local MaAsLin (version 0.0.2, downloaded from <https://bitbucket.org/biobakery/maaslin/>) in R (version 3.2.2). MaAsLin is a multivariate statistical framework that identifies associations between continuous or factor clinical metadata and microbial community abundances. Briefly, the percentage of each OTU was arcsine-square-root transformed. For each analysis of the relationship between metadata (as predictors) and transformed OTU abundance (as response variable), we included confounders (such as age, gender, Bristol stool scale and geographic location(4-6)) as covariates in the model. To further reduce the computational load, OTUs that were present in less than 10% of the population were excluded, which left 930 OTUs, constituting 96% of the initial abundance, for the following analyses. The Benjamini and Hochberg method was used for adjusting p values for multiple hypotheses (q value). In our population, 2429, 1616 and 1243 significant associations were determined at the commonly applied q-value thresholds of 0.25, 0.10 and 0.05, respectively; for our subsequent investigations, we applied the most rigorous threshold (0.05). To exclude the effects of pre-/pro-/sym-biotics on *Lactobacillus* and *Bifidobacterium*, we included pre-/pro-/sym-biotic use as confounders and repeated the multivariate association analysis (also discussed in Supplementary Results; see lines 202 to 227 in this file). We also used MaAsLin to explore the associations between gut microbes and host economic status, and we included age, gender and Bristol stool scale as confounders in this analysis.

To test the prevalence of MetS in subjects of differing economic status, we quartilised our participants according to their yearly spending (with 0-25% representing low spending, 25-75% representing moderate spending, and 75-100% representing high spending). We compared MetS prevalence among groups using a chi-square test. Multiple comparisons were adjusted by FDR corrections, and q<0.05 was considered statistically significant.

We formulated an MetS index to represent the overall gut dysbiosis related to MetS. The index is calculated as

$$MetS Index=\sum_{k=1}^{n} \log_{10} \left( {Abundance}_{k}\times10000 \right)\times{Coefficient}_{k} \times\log_{10} Q_{k}^{-1}$$

where *Abundance* is the relative abundance of an OTU, and *Coefficient* and *Q* are from the MaAsLin results presented in Additional file: Table S1. Only those OTUs that were significantly correlated with MetS were used in the calculation.

To examine the MetS prevalence within subgroups of our population, we quartilised our participants according to their MetS index value and sedentary time (with 0-25% referred to as low or quartile 1, and 75%-100% referred to as high or quartile 4). We compared MetS prevalence between different subgroups using a chi-square test. Multiple comparisons were adjusted by FDR corrections, and q<0.25 was considered statistically significant.

To test whether co-abundant groups of OTUs (guilds) had stronger correlations with MetS than did single OTUs, we applied weighted gene co-expression network analysis (WGCNA, R package WGCNA 1.61)(7) to the OTU arcsine-square root-transformed data. Briefly, we considered a signed network and selected the minimal beta value satisfying the scale-free topology criteria (optimal beta=3). The parameters for the dynamic tree cut function were deepSplit=2 and minModuleSize=10(8). Then, MaAsLin analysis was applied to these guilds to calculate their correlations with MetS and its diagnostic factors, with age, gender, Bristol stool scale and geographic locations included as confounders.

To test whether different clustering methods yielded different results, we used uclust in QIIME to generate OTUs at the 97% sequence similarity level, and we used deblur, implemented in QIIME, to generate sequence-level OTUs (sub-OTUs) using the default parameters.

**SUPPLEMENTARY RESULTS**

**Descriptions of associations between OTUs and metabolic disorders**

One hundred twenty-nine Bacteroidetes OTUs exhibited 362 associations with MetS and its diagnostic factors, including MetS (53- and 3+), waist (112- and 3+), TG (52- and 3+), SBP (56- and 1+), DBP (45- and 1+), FBG (11- and 1+) and HDL (0- and 21+). Within Bacteroidetes, the order Bacteroidales (350- and 3+) contributed to almost all of the associations. Within Bacteroidales, the associations were divided into *Bacteroides* (237- and 2+), *Parabacteroides* (36- and 0+), Rikenellaceae (38- and 0+), *Butyricimonas* (18- and 0+), *Odoribacter* (8- and 0+) and *Prevotella* (7- and 1+).

In our population, Bacteroidetes OTUs were the major group of OTUs negatively correlated with metabolic diseases. OTUs from this phylum contributed the largest number of negative associations with metabolic disorders, and the accumulated abundances of these OTUs were among the highest of all taxa. Numerous studies have reported reduced proportions of this taxonomic group in metabolic disorders(9, 10), but such reductions in relative abundance are not consistent(11). *Bacteroides* and *Prevotella* from Bacteroidetes are usually dominant at the genus level, which has been correlated with long-term diet habits(12). *Prevotella* has been suggested to be correlated with diets rich in carbohydrates or vegetables, whereas *Bacteroides* is associated with diets rich in protein and animal fats. There are no data showing that *Prevotella* has a lower association with metabolic disease, which should be expected based on the correlations of the two genera with dieting habits. However, our present results demonstrated that OTUs from both *Bacteroides* and *Prevotella* were negatively correlated with metabolic disorders, indicating that the classification of the gut microbiome dominated by these two genera might not be informative in understanding metabolic disease. In our population, in addition to *Bacteroides* OTUs, OTUs from *Parabacteroides* and *Rikenellaceae* were negatively correlated with metabolic disorders. Members within these three lineages have been reported to metabolize polysaccharides, produce acetate and propionate and increase butyrate levels through cross-feeding with other butyrate-producing taxa(13-15), thus producing a protective effect against metabolic disorders(16, 17). Despite the many previous reports, we believe that members of Bacteroidetes remain under-evaluated with respect to their potential in translational medicine relative to their evaluation with respect to their major contributions in metabolic diseases.

Firmicutes OTUs were associated with MetS (64- and 37+), waist (63- and 93+), TG (88- and 73+), SBP (33- and 25+), DBP (25- and 17+), FBG (10- and 12+) and HDL (15- and 1+). MetS-associated Firmicutes OTUs mainly came from the orders Clostridiales (282- and 229+), Erysipelotrichales (2- and 13+) and Lactobacillales (0- and 25+). Clostridiales contributed most of the associations within Firmicutes, including both negative and positive associations with MetS and its diagnostic factors. Its negative associations (referred to as F-1) mainly came from the Ruminococcaceae (176- and 30+) and Christensenellaceae (8- and 0+) families and the genera *Oscillospira* (34- and 0+), *Faecalibacterium* (14- and 2+), and *Christensenella* (4- and 0+). Positive associations with MetS and its diagnostic factors in Clostridiales (referred to as F-2) were mainly from the families of Lachnospiraceae (43- and 120+), Clostridiaceae (5- and 29+), Veillonellaceae (5- and 14+) and Peptostreptococcaceae (2- and 25+). These associations were further distributed among the genera *Blautia* (0- and 30+), [*Ruminococcus*] (0- and 22+), *Coprococcus* (7- and 10+), *Dorea* (2- and 11+), *Roseburia* (5- and 0+), *Anaerostipes* (4- and 0+), *Clostridium* (2- and 6+), *SMB53* (0- and 5+), *Megamonas* (0- and 5+), *Megasphaera* (0- and 5+) and *Dialister* (0- and 2+). Associations with MetS from the order Lactobacillales mainly came from *Lactobacillus* (0- and 6+) and *Streptococcus* (0- and 12+). Enrichment of *Lactobacillus* in MetS was unlikely due to pre-/pro-/sym-biotics consumption according to our additional analysis (Additional file 1: Supplementary Results (lines 202 to 227), Additional file 2: Table S2).

According to the analyses in the present study, the members of Firmicutes should be divided into two groups according to their roles in metabolic disorders. One group mainly includes Ruminococcaceae, which harbours important butyrate producers, such as *Faecalibacterium(18)*, and Christensenellaceae, which were reported to be genetically heritable in a British population and an anti-obesity bacterial family(19). OTUs from this group tended to be negatively correlated with metabolic disease. OTUs from *Oscillospira* contributed to the largest number of negative associations within Firmicutes, which agrees with previous reports in a UK twins cohort that this bacterial taxon showed the highest negative correlation with visceral fat(20). OTUs from the other group, which includes all of the Firmicutes taxa other than Ruminococcaceae and Christensenellaceae, tended to be positively correlated with metabolic disorders. OTUs from Lachnospiraceae had the largest number of positive associations with metabolic disorders. According to previous reports, members of this group are thought to contribute to diabetes development in germ-free mice, likely by assisting in the translocation of lipopolysaccharides through the gut barrier(21). Because Firmicutes is highly abundant and prevalent in Western populations, this phylum has been intensively studied. Many novel probiotics and treatments targeting this phylum are currently being studied(18). Our data provide insights that can aid the selection of intervention targets and the mining of potential beneficial microbes.

Ninety-six of the 137 analysed Proteobacteria OTUs were significantly associated with MetS or its diagnostic factors. These OTUs contributed 230 positive and 21 negative associations, including associations with MetS (1- and 61+), waist (4- and 68+), TG (5- and 64+), SBP (0- and 14+), DBP (0- and 7+), FBG (0- and 16+) and HDL (0- and 11+). The associations between Proteobacteria OTUs and MetS involved diverse groups, including alpha-Proteobacteria (0- and 78+), beta-Proteobacteria (3- and 49+), gamma-Proteobacteria (13- and 100+) and delta-Proteobacteria (5- and 3+). The family Enterobacteriaceae contributed 66 positive associations, which was the highest number of associations among the Proteobacteria families.

**Effects of the high level of Proteobacteria on the analysis of gut microbiome signatures of MetS**

The high level of Proteobacteria in this study was not caused by the sample collection, transportation and storage, which has been discussed previously(1). To evaluate whether our results were strongly affected by the high level of Proteobacteria, even if the high level was the result of blooms, we investigated the effects of removing Proteobacteria from our analyses. One major finding of this study is that gut microbial dysbiosis was similar across eastern and western areas; e.g., F/B, *Blautia* and gamma-Proteobacteria were increased in MetS, whereas *Bacteroides*, *Parabacteroides*, Rikenellaceae, *Faecalibacterium*, *Roseburia*, *Oscillospira*, Christensenellaceae and *Akkermansia* were decreased in MetS. First, we removed 1800 individuals who had a Proteobacteria abundance in excess of 20% in their gut from our dataset and re-performed the MaAsLin analysis. Second, we removed Proteobacteria from our dataset and re-performed the MaAsLin analysis. Both analyses revealed similar dysbiotic patterns as those obtained in our original analysis (Additional file 2: Table S6-7). As a result, we believe that the analysis of microbiome dysbiotic patterns in our study was robust despite the high level of Proteobacteria.

**Effects of medication and non-metabolic syndrome disease state on the analysis of gut microbiome signatures of MetS**

We did not exclude participants who took antibiotics, pre-/pro-/sym-biotics or medications before sampling from the analysis. However, subjects were requested to report in the questionnaire whether they had taken any of the following: antibiotics within one month before the investigation, pre-/pro-/sym-biotics within 2 weeks of starting the study, medication as directed to control blood pressure, medication when hypertension symptoms occurred, oral medication to control blood glucose and medication to control blood lipid. Participants were also requested to report whether they had IBD, IBS, colorectal carcinoma or rheumatoid arthritis.

To evaluate if any of these ten potential confounders (consumption of pre-/pro-/sym-biotics, administration of antibiotics, medication as directed to control blood pressure, medication when hypertension symptoms occurred, oral medication to control blood glucose, medication to control blood lipid, IBD, IBS, colorectal carcinoma and rheumatoid arthritis) affected our determination of MetS-correlated organisms, two analyses were conducted. First, we added these ten variables as confounders, together with age, gender, Bristol stool scale and geographic location, and re-performed the MaAsLin (Additional file 2: Table S8). Second, we removed the 1819 individuals who were positive for any of the ten variables and re-performed the MaAsLin (Additional file 2: Table S9). Both analyses generated correlation patterns similar to those obtained in our original analysis; e.g., most OTUs from Bacteroidetes, Ruminococcaceae, Christensenellaceae and *Akkermansia* were negatively correlated with MetS, whereas those from Actinobacteria, Firmicutes (other than Ruminococcaceae or Christensenellaceae) and Proteobacteria were positively correlated with MetS. Taken together, our analyses indicated that the results reported in the main text were not biased by medication and non-metabolic syndrome disease state.

**Associations between co-abundant OTU groups or sub-OTUs and MetS**

The coefficient calculated by MaAsLin was small due to the zero-inflation problem of the microbiota data. We investigated whether co-abundant groups of OTUs (guilds) or sub-OTUs (sequence-level OTUs)(22) yielded higher coefficients than did individual 97%-similarity OTUs. To generate co-abundant OTU groups, we applied weighted gene co-expression network analysis (WGCNA)(7) to the OTU arcsine-square root-transformed data, and 14 guilds were generated. OTUs from the same taxa tended to be clustered in one guild. The 14 guilds comprised four Bacteroidetes guilds (guilds that mainly contained Bacteroidetes OTUs), three Proteobacteria guilds, three F-1 (Ruminococcaceae and Christensenellaceae) guilds, three F-2 (Firmicutes other than Ruminococcaceae and Christensenellaceae) guilds and one Bacteroidetes & Firmicutes guild (Additional file 2: Table S10). Then, MaAsLin analysis was applied to these 14 guilds to calculate their correlations with MetS and its diagnostic factors. Age, gender, Bristol stool scale and geographic location were used as confounders. The results showed that all four Bacteroidetes guilds and one F-1 guild were negatively correlated with MetS, whereas two F-2 guilds and two Proteobacteria guilds were positively correlated with MetS. These results agree with the OTU-level MaAsLin analysis. However, the grouping of OTUs into guilds did not improve the coefficients of correlation with MetS. The highest negative correlation coefficient was -0.029 (a Bacteroidetes guild, q=1.72*10^-6^), whereas the highest positive correlation coefficient was 0.042 (a Proteobacteria guild, q=1.99*10^-8^), which were comparable to those of single OTUs. Similar results were achieved when calculating the associations between deblur(22)-generated sub-OTUs and MetS by MaAsLin using the same parameters and confounders (Additional file 2: Table S11). Sub-OTUs from Bacteroidetes and Ruminococcaceae tended to correlate negatively with MetS, whereas those from Proteobacteria and Firmicutes other than Ruminococcaceae tended to correlate positively with MetS. Moreover, among the sub-OTUs, one sub-OTU classified as *Akkermansia muciniphila* showed the highest negative correlation coefficient (coefficient=-0.018, q=5.57*10^-4^), and one sub-OTU classified as *E. coli* showed the highest positive correlation coefficient (coefficient=0.043, q=1.52*10^-7^). These results indicated that the observed dysbiotic patterns of MetS in the present population were consistent across different calculation methods. We also tested a traditional clustering method, uclust, and the results agreed with the above association patterns (Table S12).

**Differences in OTU mean abundance between MetS and non-MetS subjects largely corresponded to MaAsLin coefficients**

To obtain an intuitive understanding of the MaAsLin coefficients, we analysed the differences in OTU mean abundance between MetS and non-MetS subjects by Wilcoxon rank sum test (Table S13). The results showed that most of the OTUs that were found to significantly differ between the two groups in the MaAsLin analysis also showed significant differences in the Wilcoxon rank sum test and that higher coefficients corresponded to greater differences in OTU abundance.

**SUPPLEMENTARY FIGURES**

**
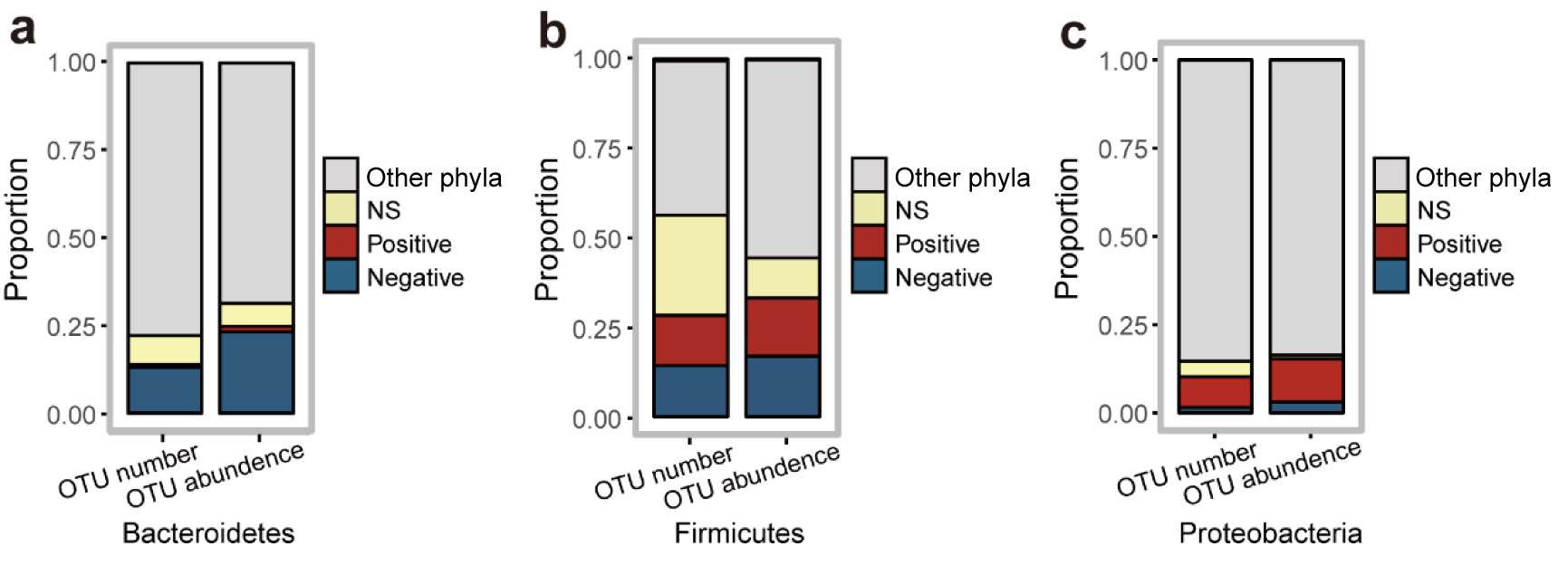
**

Figure S1. Proportions of OTUs associated with MetS in terms of OTU number and their accumulated abundance within (**a**) Bacteroidetes, (**b**) Firmicutes, and (**c**) Proteobacteria.


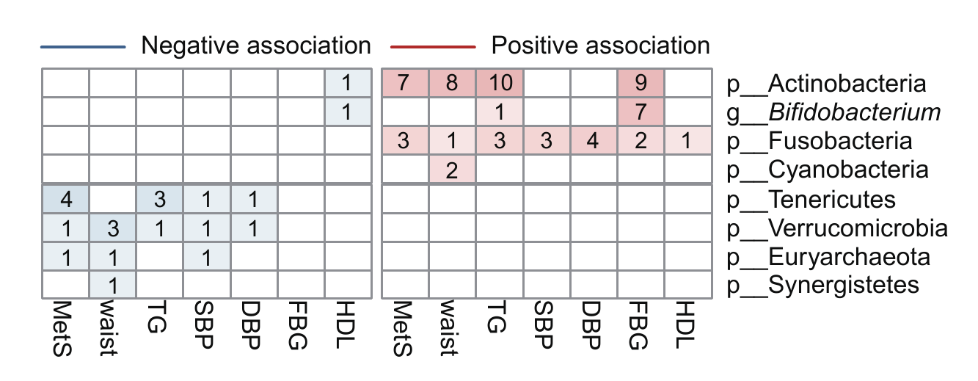


Figure S2. Number of associations between MetS and low-abundance taxa.


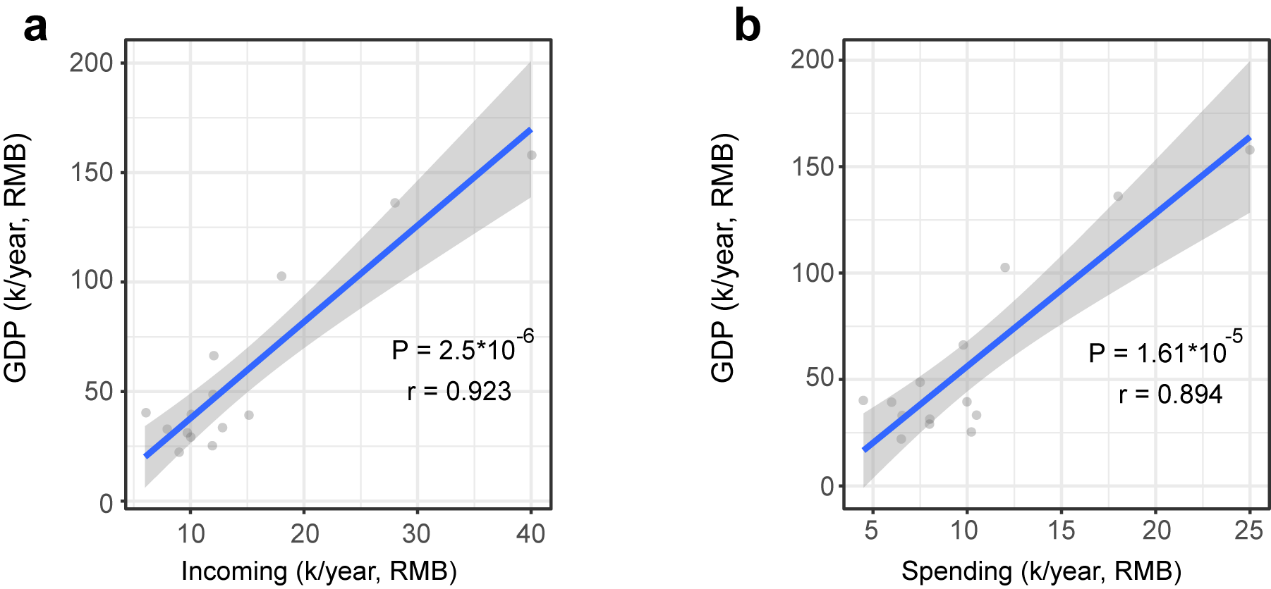


Figure S3. Correlations between local GDP per capita and average yearly income (a) and spending (b) as determined by Spearman’s rank correlation test.

**References for Supplementary Data**

1. He Y, Wu W, Zheng H-M, Li P, McDonald D, Sheng H-F, et al. Regional variation limits applications of healthy gut microbiome reference ranges and disease models. Nature Medicine. 2018 DOI: 10.1038/s41591-018-0164-x.

2. Morgan XC, Tickle TL, Sokol H, Gevers D, Devaney KL, Ward DV, et al. Dysfunction of the intestinal microbiome in inflammatory bowel disease and treatment. Genome Biol. 2012;13(9):R79.

3. Alexandra Zhernakova AK, Marc Jan Bonder, Ettje F. Tigchelaar, Melanie Schirmer, Tommi Vatanen, Zlatan Mujagic, Arnau Vich Vila, Gwen Falony, Sara Vieira-Silva, Jun Wang, Floris Imhann, Eelke Brandsma, Soesma A. Jankipersadsing, Marie Joossens, Maria Carmen Cenit, Patrick Deelen, Morris A. Swertz, LifeLines cohort study, K. Weersma, Edith J. M. Feskens, Mihai G. Netea, Dirk Gevers, Daisy Jonkers, Lude Franke, Yurii S. Aulchenko, Curtis Huttenhower, Jeroen Raes, Marten H. Hofker, Ramnik J. Xavier, Cisca Wijmenga, Jingyuan Fu. Population-based metagenomics analysis reveals markers for gut microbiome composition and diversity. Science. 2016;352(6285):5.

4. Falony G, Joossens M, Vieira-Silva S, Wang J, Darzi Y, Faust K, et al. Population-level analysis of gut microbiome variation. Science. 2016;352(6285):560-4.

5. Xie H, Guo R, Zhong H, Feng Q, Lan Z, Qin B, et al. Shotgun Metagenomics of 250 Adult Twins Reveals Genetic and Environmental Impacts on the Gut Microbiome. Cell Syst. 2016;3(6):572-84 e3.

6. Zhernakova A, Kurilshikov A, Bonder MJ, Tigchelaar EF, Schirmer M, Vatanen T, et al. Population-based metagenomics analysis reveals markers for gut microbiome composition and diversity. Science. 2016;352(6285):565-9.

7. Langfelder P, Horvath S. WGCNA: an R package for weighted correlation network analysis. BMC Bioinformatics. 2008;9:559.

8. Langfelder P, Zhang B, Horvath S. Defining clusters from a hierarchical cluster tree: the Dynamic Tree Cut package for R. Bioinformatics. 2008;24(5):719-20.

9. Turnbaugh PJ, Ley RE, Mahowald MA, Magrini V, Mardis ER, Gordon JI. An obesity-associated gut microbiome with increased capacity for energy harvest. Nature. 2006;444(7122):1027-31.

10. Ridaura VK, Faith JJ, Rey FE, Cheng J, Duncan AE, Kau AL, et al. Gut microbiota from twins discordant for obesity modulate metabolism in mice. Science. 2013;341(6150):1241214.

11. Le Chatelier E, Nielsen T, Qin J, Prifti E, Hildebrand F, Falony G, et al. Richness of human gut microbiome correlates with metabolic markers. Nature. 2013;500(7464):541-6.

12. Wu GD, Chen J, Hoffmann C, Bittinger K, Chen YY, Keilbaugh SA, et al. Linking long-term dietary patterns with gut microbial enterotypes. Science. 2011;334(6052):105-8.

13. Mahowald MA, Rey FE, Seedorf H, Turnbaugh PJ, Fulton RS, Wollam A, et al. Characterizing a model human gut microbiota composed of members of its two dominant bacterial phyla. Proceedings of the National Academy of Sciences of the United States of America. 2009;106(14):5859-64.

14. Duncan SH, Holtrop G, Lobley GE, Calder AG, Stewart CS, Flint HJ. Contribution of acetate to butyrate formation by human faecal bacteria. The British journal of nutrition. 2004;91(6):915-23.

15. Vanessa K. Ridaura JJF, Federico E. Rey, Jiye Cheng, Alexis E. Duncan, Andrew L. Kau, Nicholas W. Griffin, Vincent Lombard, Bernard Henrissat, James R. Bain, Michael J. Muehlbauer, Olga Ilkayeva, Clay F. Semenkovich, Katsuhiko Funai, David K. Hayashi, Barbara J. Lyle, Margaret C. Martini, Luke K. Ursell, Jose C. Clemente, William Van Treuren, William A. Walters, Rob Knight, Christopher B. Newgard, Andrew C. Heath, Jeffrey I. Gordon. Gut Microbiota from Twins Discordant for Obesity Modulate Metabolism in Mice. Science. 2013;341.

16. Gao Z, Yin J, Zhang J, Ward RE, Martin RJ, Lefevre M, et al. Butyrate improves insulin sensitivity and increases energy expenditure in mice. Diabetes. 2009;58(7):1509-17.

17. Lin HV, Frassetto A, Kowalik EJ, Jr., Nawrocki AR, Lu MM, Kosinski JR, et al. Butyrate and propionate protect against diet-induced obesity and regulate gut hormones via free fatty acid receptor 3-independent mechanisms. PloS one. 2012;7(4):e35240.

18. Barcenilla A, Pryde SE, Martin JC, Duncan SH, Stewart CS, Henderson C, et al. Phylogenetic relationships of butyrate-producing bacteria from the human gut. Appl Environ Microbiol. 2000;66(4):1654-61.

19. Goodrich Julia K, Waters Jillian L, Poole Angela C, Sutter Jessica L, Koren O, Blekhman R, et al. Human Genetics Shape the Gut Microbiome. Cell. 2014;159(4):789-99.

20. Beaumont M, Goodrich JK, Jackson MA, Yet I, Davenport ER, Vieira-Silva S, et al. Heritable components of the human fecal microbiome are associated with visceral fat. Genome Biol. 2016;17(1):189.

21. Kameyama K, Itoh K. Intestinal colonization by a Lachnospiraceae bacterium contributes to the development of diabetes in obese mice. Microbes Environ. 2014;29(4):427-30.

22. Amir A, McDonald D, Navas-Molina JA, Kopylova E, Morton JT, Zech Xu Z, et al. Deblur Rapidly Resolves Single-Nucleotide Community Sequence Patterns. mSystems. 2017;2(2).
